# Primary myelofibrosis: spectrum of imaging features and disease-related complications

**DOI:** 10.1186/s13244-019-0758-y

**Published:** 2019-08-07

**Authors:** Sheng Fei Oon, Dalveer Singh, Teng Han Tan, Allan Lee, Geertje Noe, Kate Burbury, Joseph Paiva

**Affiliations:** 1 Department of Radiology, Peter MacCallum Cancer Centre, Melbourne, Australia; 2 Department of Haematology, Peter MacCallum Cancer Centre, Melbourne, Australia; 3 University of Melbourne, Melbourne, Australia

**Keywords:** Myeloproliferative neoplasm, Myelofibrosis, Extramedullary haematopoiesis, Oncologic imaging, Haematology

## Abstract

Primary myelofibrosis is a chronic clonal stem cell disorder that results in a build-up of marrow fibrosis and dysfunction, hypermetabolic states, and myeloid metaplasia. The clinical and radiological consequences can be quite diverse and range from the manifestations of osteosclerosis and extramedullary haematopoiesis to thrombohaemorrhagic complications from haemostatic dysfunction. In addition, there is the challenge of identifying less well-recognised sites of extramedullary haematopoiesis and their site-specific complications. The intent of this article is to illustrate the spectrum of primary myelofibrosis as declared though multimodality imaging, with examples of both common and rarer disease manifestations.

## Key points

- Classic appearances of myelofibrosis include diffuse osteosclerosis, massive splenomegaly, and extramedullary haematopoiesis.
- Thromboembolisms are a common complication of myelofibrosis and often occur in unusual areas.
- Splanchnic vein thrombosis in a young patient raises suspicion of myeloproliferative neoplasm.
- Massive splenomegaly may be complicated by spontaneous splenic infarction or haemorrhage.

## Introduction

Myelofibrosis is a chronic clonal stem cell disorder, alongside other myeloproliferative neoplasms (MPNs) such as polycythaemia vera, essential thrombocythaemia, and chronic myeloid leukaemia (CML) [1]. Myelofibrosis commonly demonstrates well-described characteristic imaging features, namely diffuse osteosclerosis, massive splenomegaly, and extramedullary haematopoiesis (EMH).

## Epidemiology, clinical features, and presentations

The annual incidence of primary myelofibrosis (PMF) is 0.4–1.4 per 100,000 population [2, 3] and shows a predilection for older males, although younger patients can be affected. Of the MPNs, PMF is the least common [4] but is associated with poorer survival, approximately 2 to 5 years upon diagnosis and symptom onset [5]. Patients may be asymptomatic and present following detection of incidental radiological findings or through discovery of anaemia, thrombocytosis, or thrombocytopaenia. In symptomatic patients, the clinical presentation varies from constitutional symptoms [4] to cardiovascular complications related to severe anaemia and thromboembolic events. Up to 10% of patients experience a thromboembolic event, most commonly venous thromboembolism [6]. Splenomegaly is an inevitable outcome and may lead to splenic infarction, haemorrhage, splanchnic vein thrombosis, portal hypertension, or mass effect symptoms [4, 7–9]. There is also a small risk of progression to acute myeloid leukaemia [10].

## Imaging

**Fig. 1:**
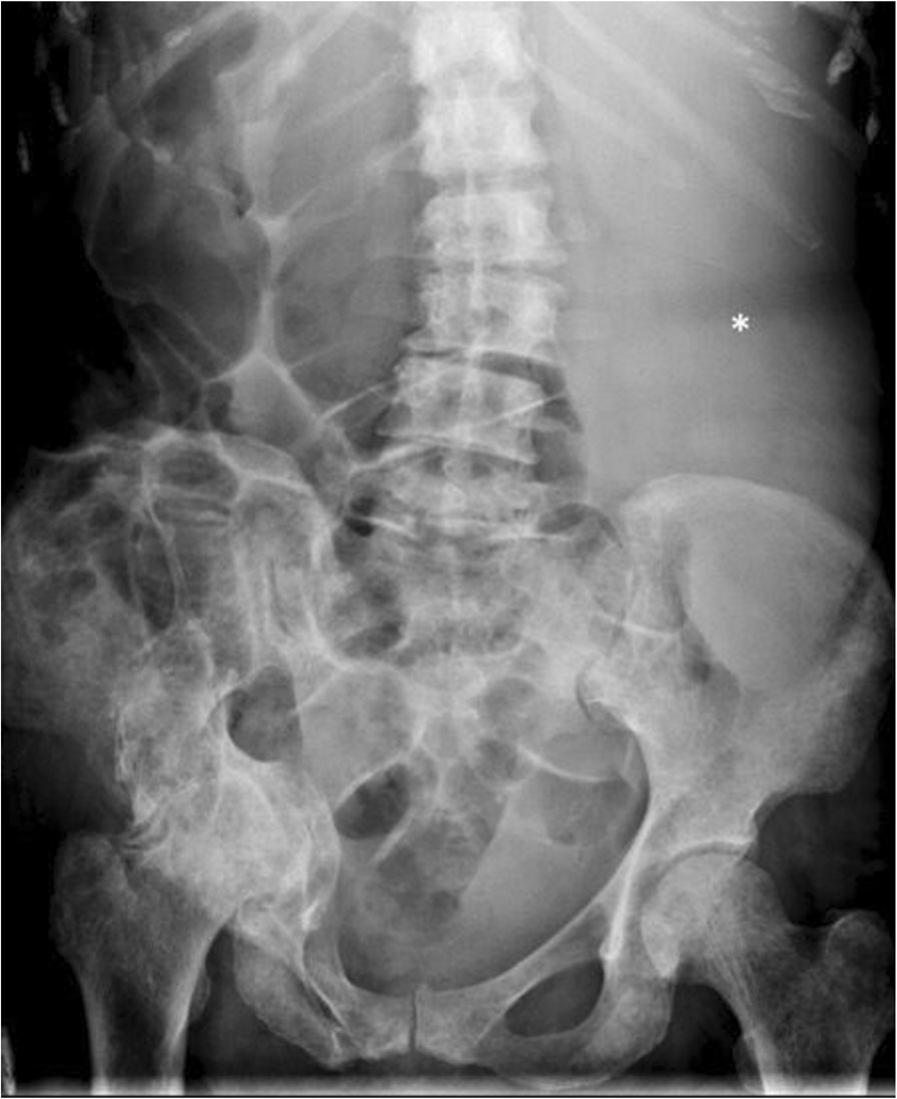
Plain abdominal radiograph demonstrating diffuse increased osteosclerosis and a large left upper quadrant shadow consistent with massive splenomegaly (asterisk) in a patient with known primary myelofibrosis. There is marked displacement of the large and small bowel to the right by the spleen

**Fig. 2:**
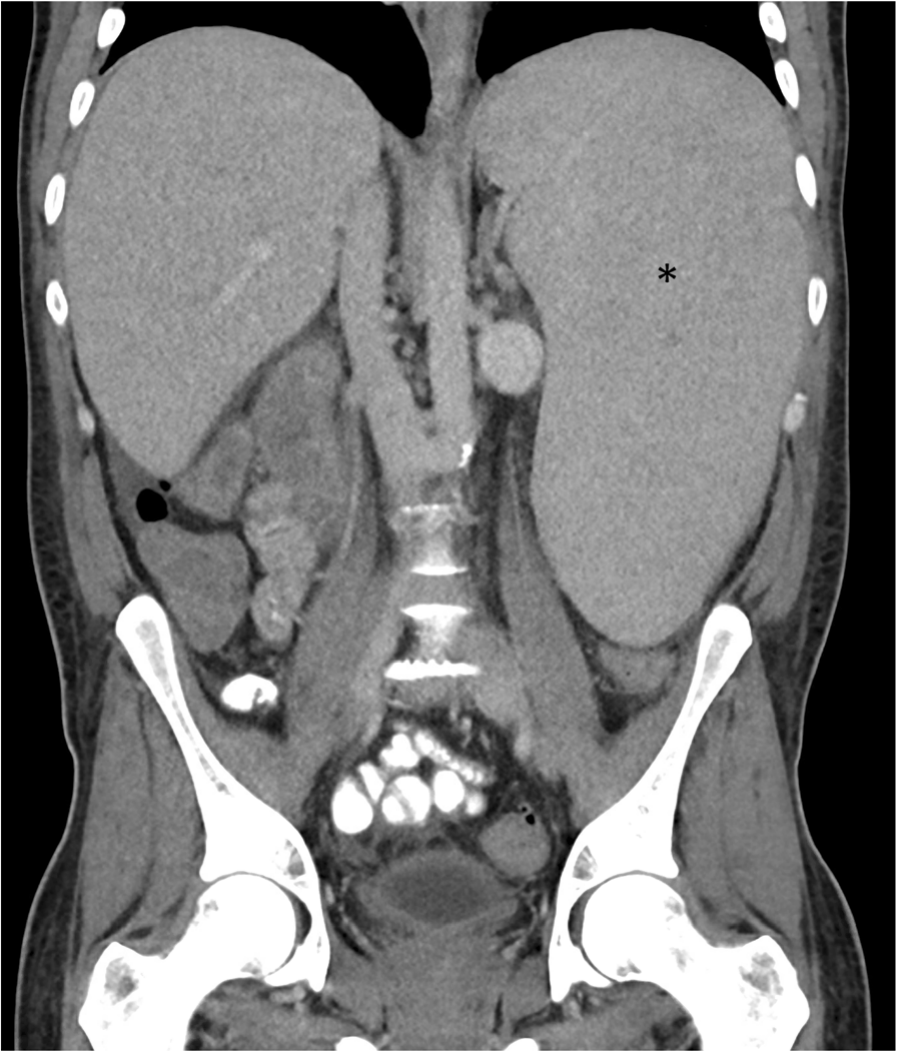
Coronal CT of the abdomen demonstrating a markedly enlarged spleen (asterisk). The combination of osteosclerosis and massive splenomegaly has a narrow differential diagnosis and is most suggestive of myelofibrosis

Classical imaging appearances of myelofibrosis include diffuse osteosclerosis which often affects the entire axial and appendicular skeleton, massive splenomegaly, and EMH (Figs. 1 and 2). This triad of appearances, however, is not exclusive to myelofibrosis and can be found in other MPN and lymphoma.

### Splenomegaly

Splenomegaly in PMF is caused by splenic EMH and is moderate to severe in about 75% of cases [4]. The dysregulation of the bone marrow microenvironment in PMF is a key feature of the disease and is characterised by the abnormal trafficking patterns of haematopoietic stem cells and haematopoietic progenitor cells. The cells migrate and are engrafted into sites external to the bone marrow, such the spleen, and expansion of the haematopoietic space from continuous proliferation of the malignant clones within the splenic microenvironment leads to progressive splenomegaly [11].

The upper limit of the spleen is defined as a craniocaudal length of 15 cm, 10 cm lateral width, or 6 cm anteroposterior dimension. The ‘splenic index’ is the product of width, depth, and length of the spleen (normal range 160–440 cm^3^) [12]. Splenomegaly is palpable in up to 90% of patients at the time of initial presentation [13], but at presentation, only about 25% are symptomatic, often with vague abdominal fullness or discomfort [4]. An abdominal radiograph may reveal an enlarged splenic opacity, which may provide a clue to underlying MPN, especially if large enough to displace the left renal opacity or bowel loops (Fig. 1). CT (Fig. 2) confirms the radiographic features of PMF, is the best modality for assessing the integrity of the spleen and vasculature, and allows for evaluation for other potential complications and PMF disease hallmarks such as EMH.

### Extramedullary haematopoiesis

EMH occurs when an inadequate production of blood cells by the bone marrow necessitates production of blood cells in other source tissues. In the foetus, the yolk sac is the primary centre of blood cell production, followed by the spleen and liver. After birth, however, blood cell production occurs primarily in the bones [14]. In conditions where the marrow is replaced by fibrosis, EMH occurs. This most commonly occurs in PMF, but other examples of conditions causing a similar process include leukaemia, sickle cell disease, and thalassaemia [15].

**Fig. 3:**
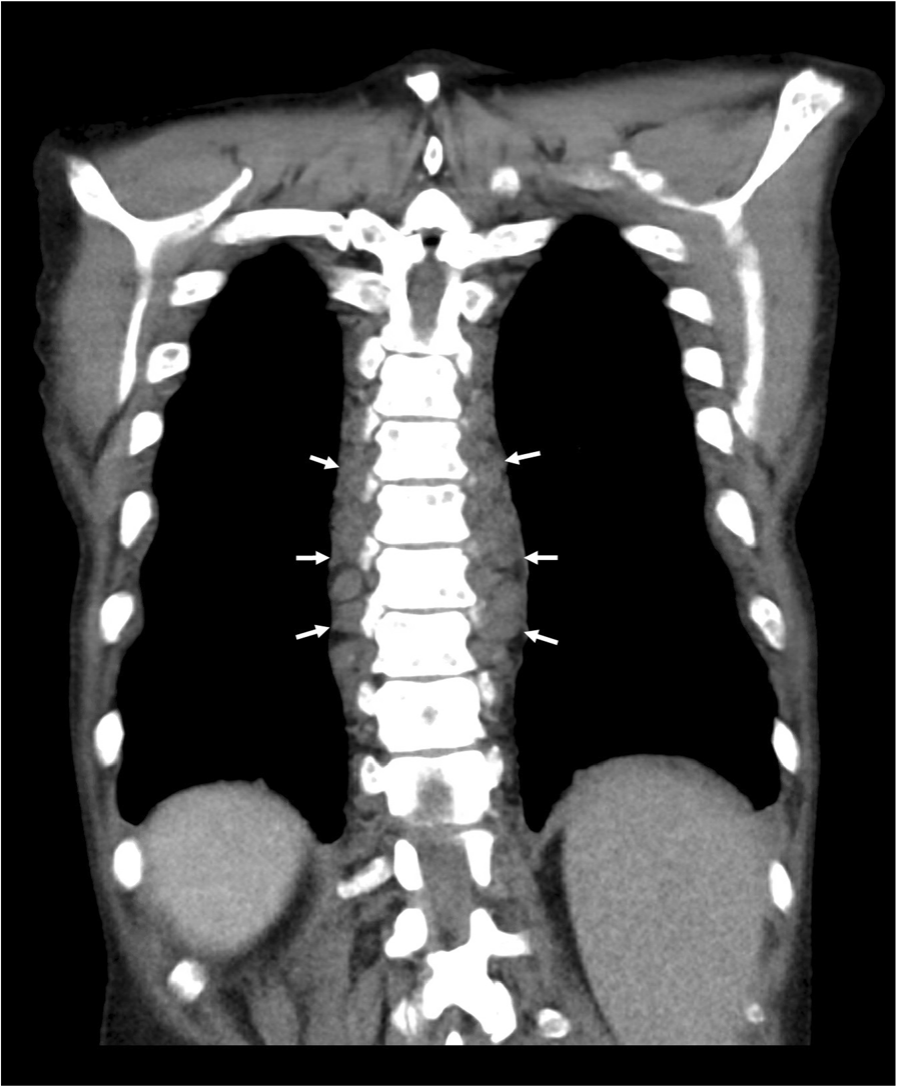
Coronal CT of the chest demonstrating extramedullary haematopoiesis in the posterior mediastinum, which typically appear as bilateral and symmetrical posterior mediastinal masses (white arrows)

**Fig. 4:**
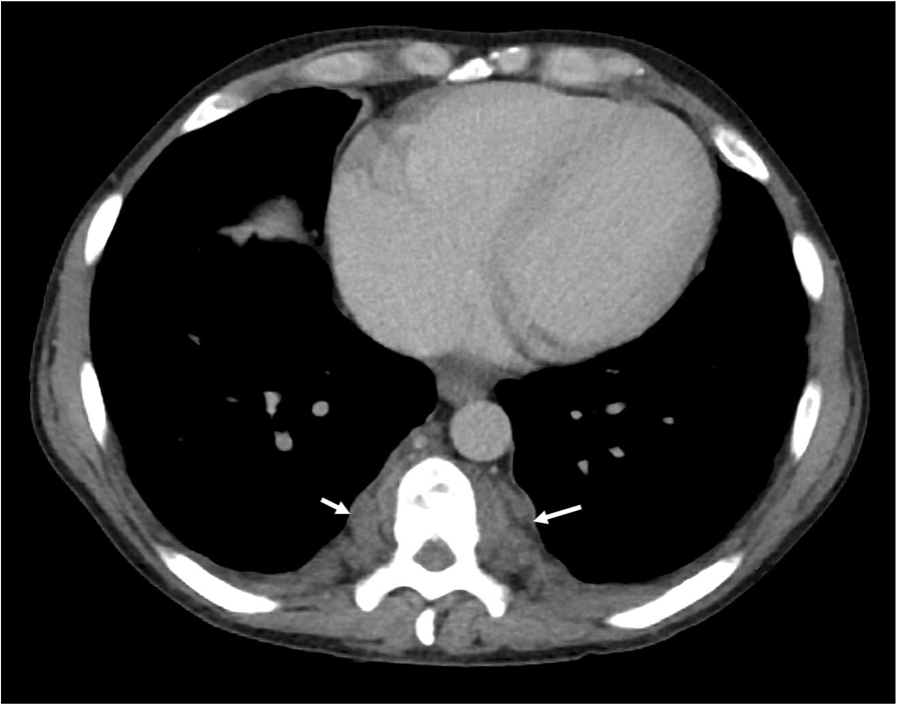
Axial CT of the chest demonstrating extramedullary haematopoiesis in the posterior mediastinum, which typically appear as bilateral and symmetrical posterior mediastinal masses (white arrows). In the paravertebral regions, the soft tissue may compress the exiting nerves in the neural exit foramina or enter the vertebral canal, causing cord displacement or compression

**Fig. 5:**
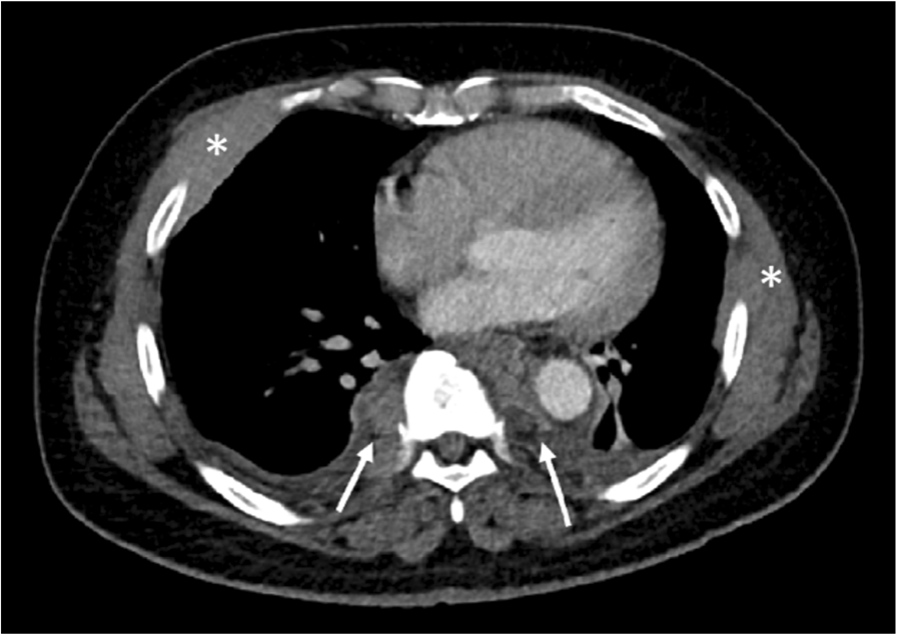
Axial CT of the chest in another patient with myelofibrosis demonstrating soft tissue masses in the left and right intercostal spaces (asterisk), proven extramedullary haematopoiesis on histology. Note the bilateral bulky posterior mediastinal masses, also extramedullary haematopoiesis (white arrows)

In the thorax, EMH is most commonly seen as posterior mediastinal or paravertebral soft tissue masses (see Figs. 3 and 4). However, this would be regarded as a rare cause with the more likely differential diagnoses for posterior mediastinal masses being lymphoma, neurogenic tumours (often associated with rib splaying), and vascular anomalies (typically unilateral) [16]. On CT images, EMH is typically bilateral, smooth, and lobulated; may contain intralesional fat; and usually does not erode the adjacent bone. MRI chemical shift imaging may be helpful in identifying microscopic fat [15]. The second commonest presentation of EMH in the thorax is rib expansion, although intercostal space lesions are also an important imaging presentation (Fig. 5). Rarely, EMH can present as pulmonary nodules, masses, or fibrosis [17].

**Fig. 6:**
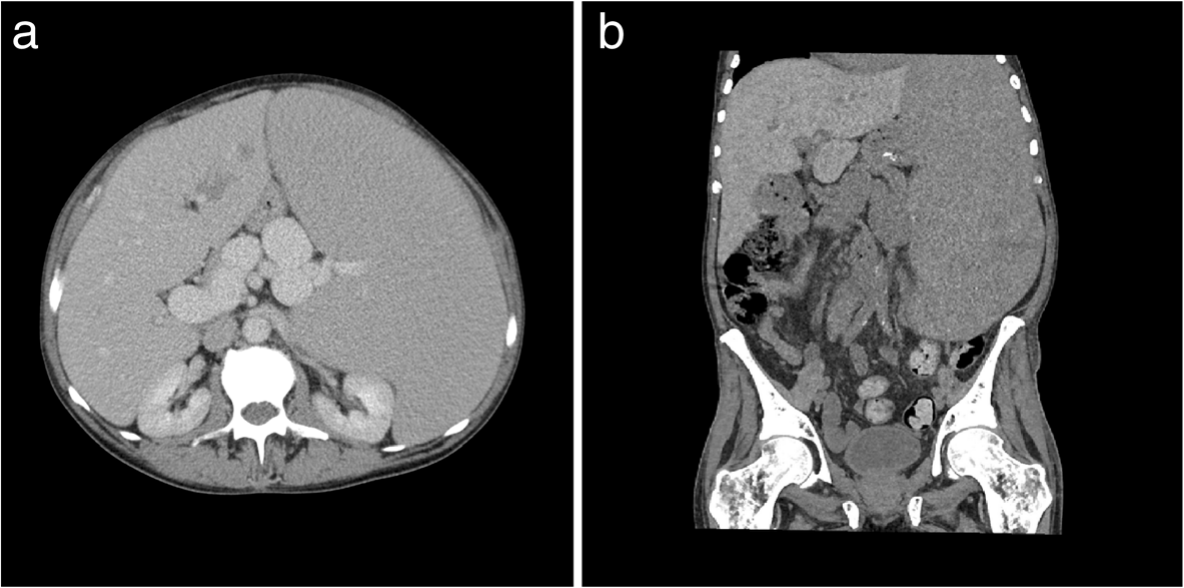
Portovenous phase axial (**a**) and coronal (**b**) CT of the abdomen in two different patients with myelofibrosis demonstrating osteosclerosis and massive splenomegaly. Note the presence of splenic varices and cavernous transformation of the portal vein on the axial image (**a**). Periportal low attenuation is also seen, consistent with periportal oedema and in keeping with portal hypertension. On the coronal image, linear low attenuation at the inferior aspect of the spleen is consistent with splenic infarction, another complication of massive splenomegaly

In the abdomen, EMH most frequently occurs in the liver, spleen, and lymph nodes. Hepatosplenomegaly is the typical manifestation and is usually evident as diffuse organomegaly without a focal mass (Fig. 6) [18]. Splenomegaly is the most common manifestation of EMH in PMF, with palpable splenomegaly evident in over 80% of patients [13, 19]. Hepatomegaly is slightly less common and occurs in 39–65% of patients at the time of presentation [19, 20]. EMH in the liver or spleen may have different appearances on MRI depending on the underlying activity status. Lesions that are inactive and long-standing, or in patients treated with blood transfusions, may decrease in size and show features of extensive iron deposition, which manifests as increased signal on out-of-phase sequences relative to in-phase sequences. Chronic inactive lesions also possess a higher proportion of fat infiltration and iron deposition and so typically appear as T1 and T2 hypointense lesions with little or no enhancement. Active haematopoietic masses, on the other hand, have a greater proportion of erythroid and myeloid cells and less fat infiltration and iron deposition, and manifest more commonly as T1 intermediate and T2 hyperintense lesions, often with some enhancement [21, 22].

**Fig. 7:**
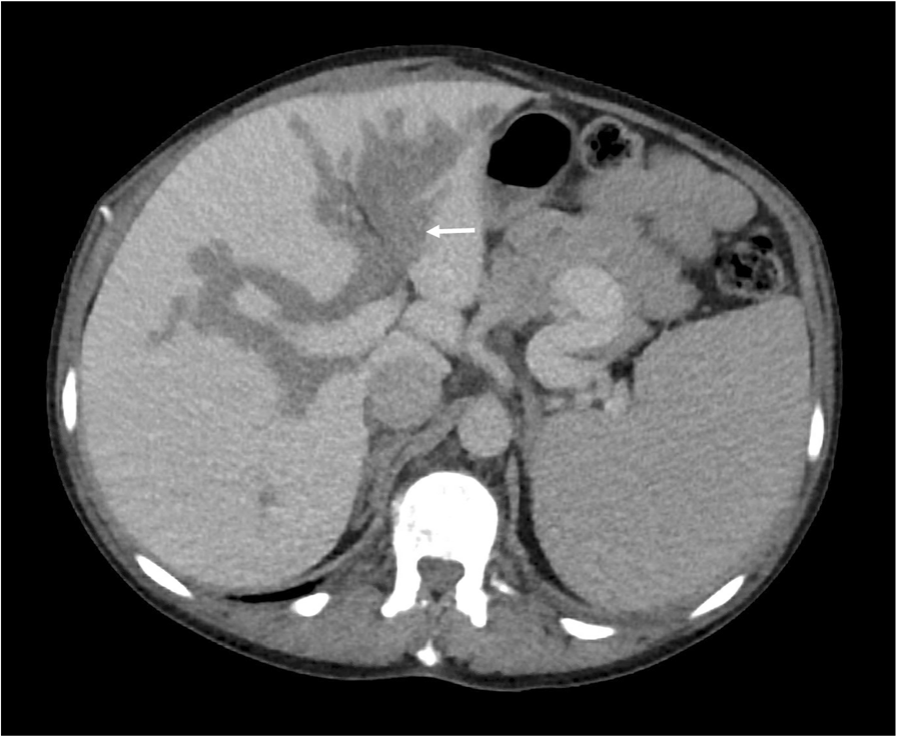
Axial CT demonstrating potential sites of extramedullary haematopoiesis (EMH) in the body. The commonest sites of involvement are the liver and spleen which manifest as hepatosplenomegaly. Less commonly, EMH may present in the periportal region (white arrows) and may be indistinguishable from periportal oedema. Clues to differentiating periportal EMH from periportal oedema include a more lobular appearance to EMH and soft tissue attenuation, whereas periportal oedema is generally less lobular and may have fluid attenuation

Periportal or peribiliary EMH can very closely resemble periportal oedema on CT, appearing as uniform low attenuation masses with clear margins. Both entities may also coexist in more advanced cases of PMF complicated by portal hypertension. A thick, lobulated appearance with soft tissue attenuation on CT may provide a differentiating factor against periportal oedema if periportal EMH is extensive (Fig. 7). On MRI, these generally appear as T1 hypointense and slightly T2 hyperintense, with heterogenously delayed enhancement [23, 24].

**Fig. 8:**
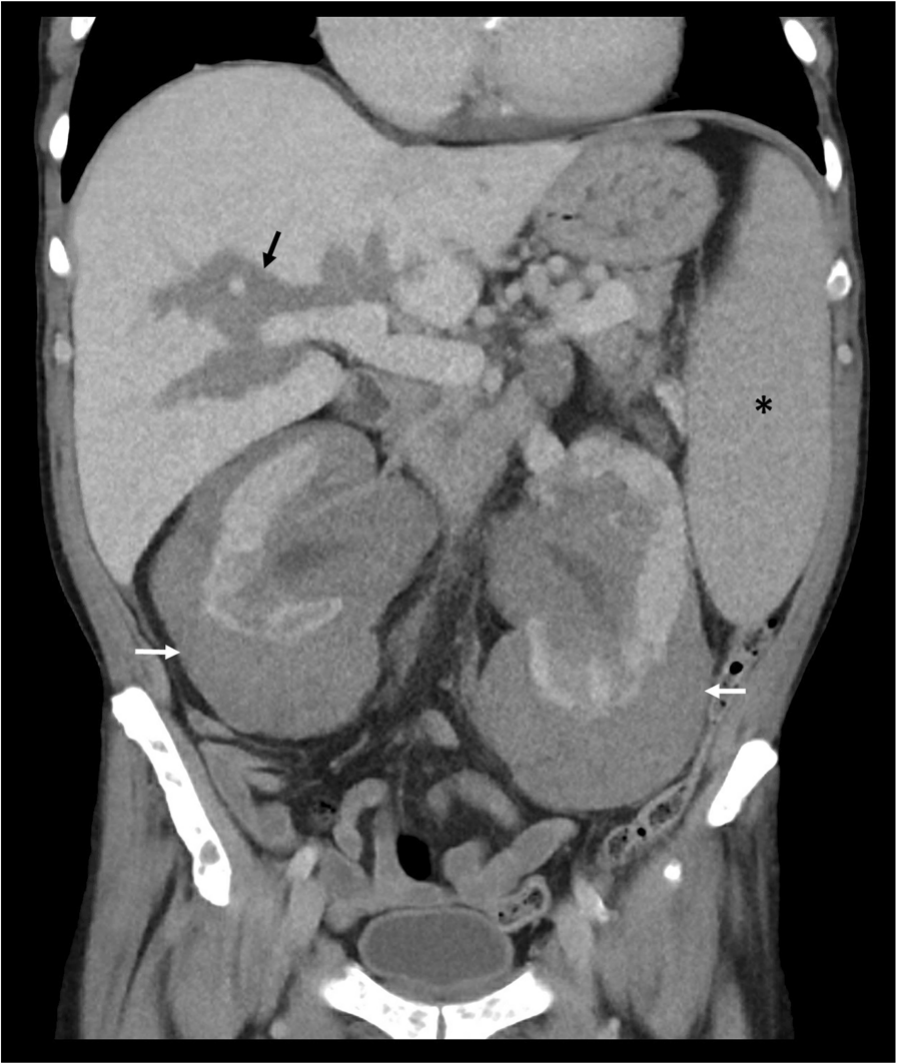
Coronal CT demonstrating bilateral lobular perirenal soft tissue masses, contained by Gerota’s fascia and not causing contour deformity against the kidneys (white arrows), consistent with perirenal extramedullary haematopoiesis, a common site of extramedullary haematopoiesis in the abdomen. Note also the presence of periportal extramedullary haematopoiesis (black arrow) and splenomegaly (asterisk) in addition to osteosclerosis

**Fig. 9:**
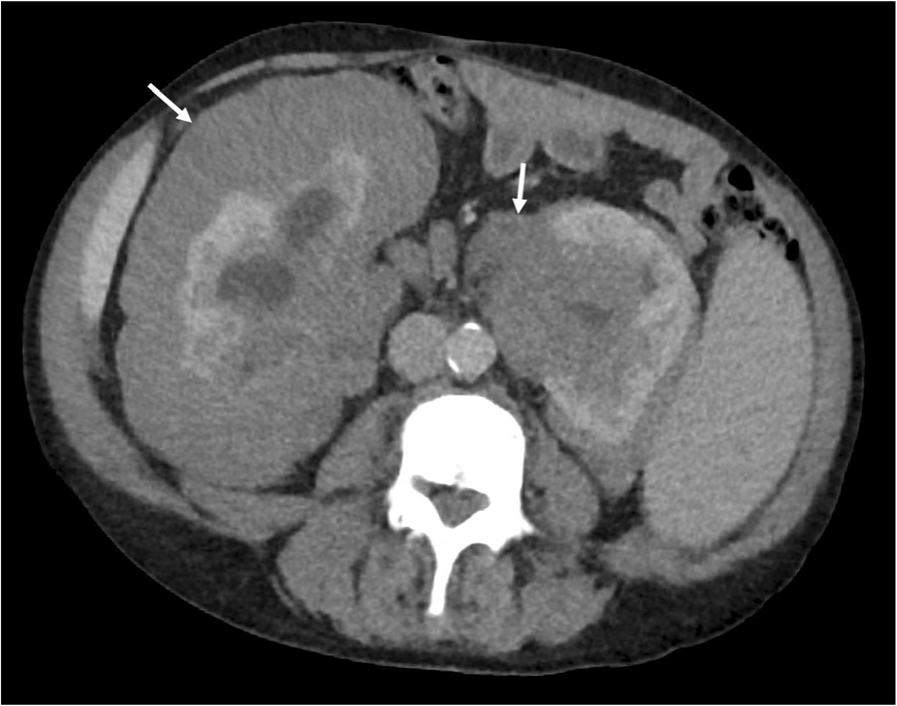
Axial CT demonstrating bilateral lobular perirenal soft tissue masses, contained by Gerota’s fascia and often not causing contour deformity against the kidneys (white arrows), consistent with perirenal extramedullary haematopoiesis, a common site of extramedullary haematopoiesis in the abdomen

Perirenal EMH is another important site and typically appears as a thick rind of low attenuation homogenous soft tissue around the kidneys, lobulated but not typically causing significant renal contour deformity (Figs. 8 and 9). The main differential diagnoses for bilateral perirenal soft tissue abnormality to this extent include renal lymphoma and Erdheim-Chester disease; however, these are only distinguishable on biopsy [15].

**Fig. 10:**
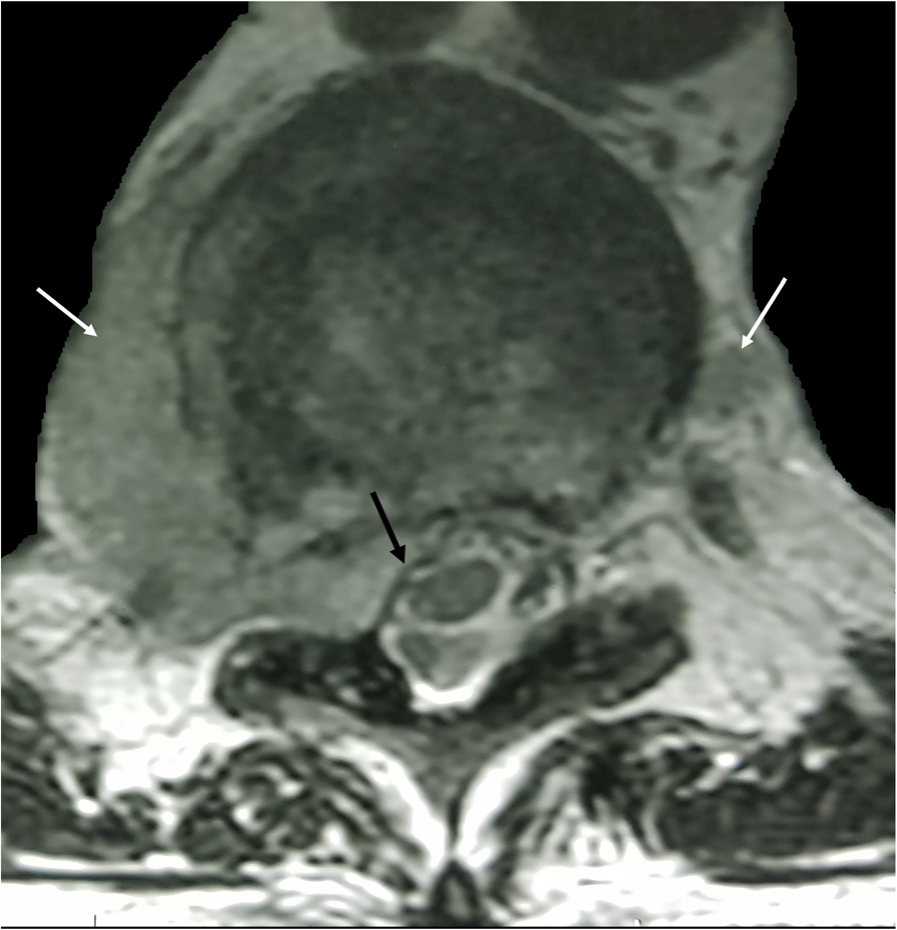
T2-weighted MRI demonstrating bilateral posterior mediastinal masses (white arrows) consistent with extramedullary haematopoiesis. The soft tissue has intruded the right neural exit foramen and into the vertebral canal, causing effacement and mild displacement of the thoracic cord (black arrow)

EMH in the central nervous system most commonly occurs as epidural soft tissue masses, within either the brain or the spine (Fig. 10). On MRI, EMH demonstrates heterogenous and variable T1 and T2 signal. Nuclear scintigraphy utilising ^99m^technetium-(Tc) sulphur colloid can assist in confirming EMH by identifying bone marrow elements within the masses [15].

**Fig. 11:**
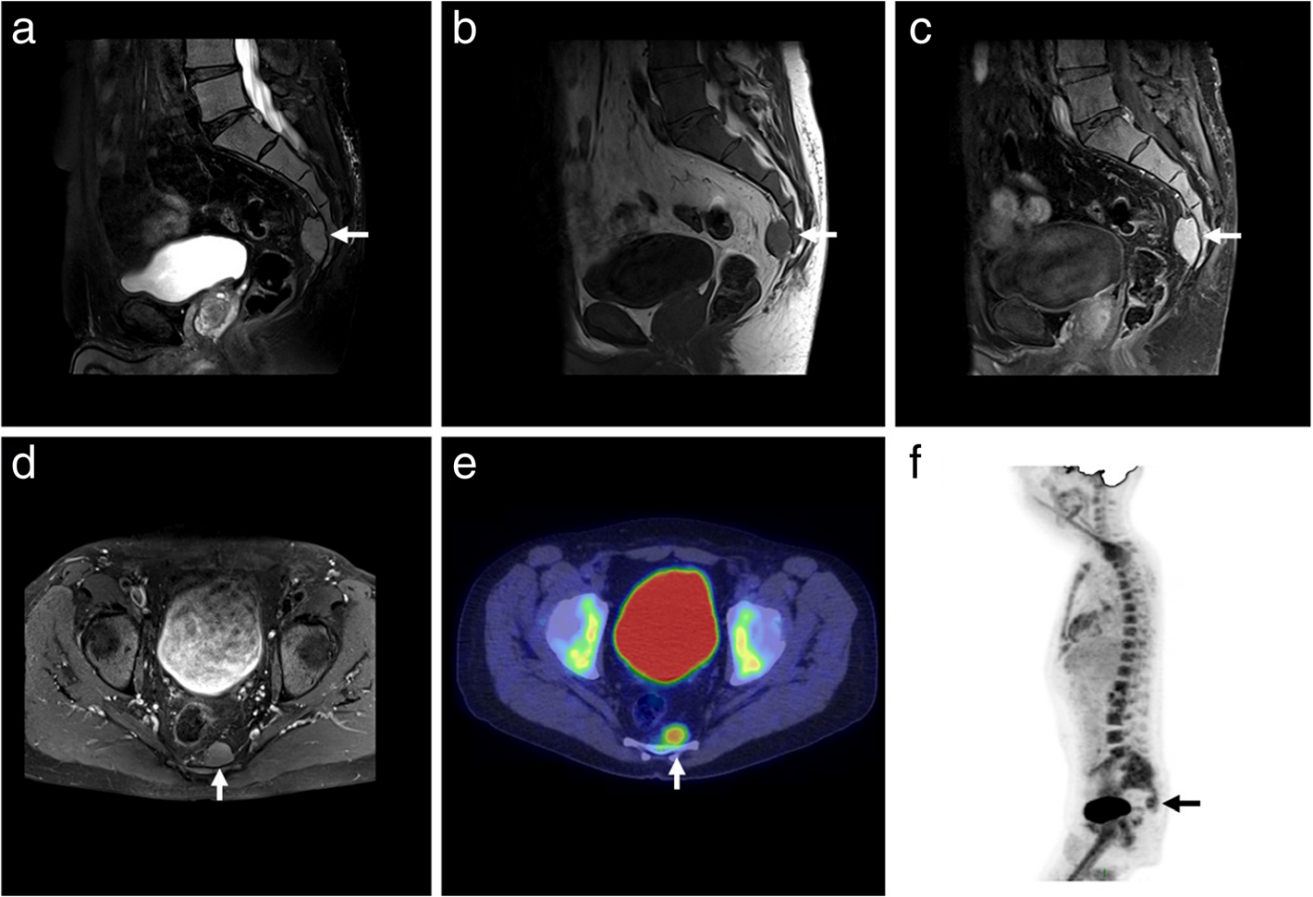
Presacral extramedullary haematopoiesis. **a** Sagittal. **b** T2. **c** Pre-contrast T1. **d** Post-contrast T1FS. **e** Axial post-contrast T1FS. **f** Fusion PET/CT with colour map. FDG-18 PET/CT MIP reconstruction demonstrating a presacral lesion with increased FDG uptake, consistent with presacral extramedullary haematopoiesis. Note the ‘superscan’ appearance from diffuse FDG uptake in the axial and appendicular skeleton (**f**)

**Fig. 12:**
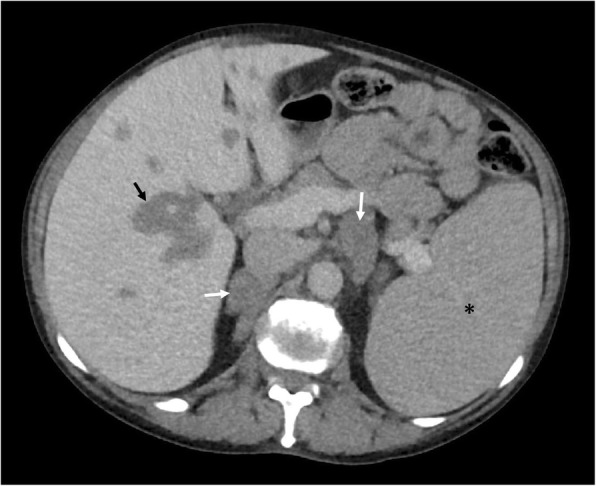
Axial CT of the abdomen demonstrates symmetrical bulky and homogenous low attenuating masses in the adrenal glands (white arrows), consistent with extramedullary haematopoiesis in the adrenal glands. Note also similar attenuation bulky perihilar soft tissue consistent with periportal extramedullary haematopoiesis and splenomegaly (asterisk)

Other rare sites of EMH involvement include the presacral region (Fig. 11), adrenal glands (Fig. 12), nasopharynx, paranasal sinuses, gastrointestinal or urinary tract, prostate, peritoneum, skin, breast, middle ear, lacrimal glands, and omentum [25, 26].

### Osteosclerosis

**Fig. 13:**
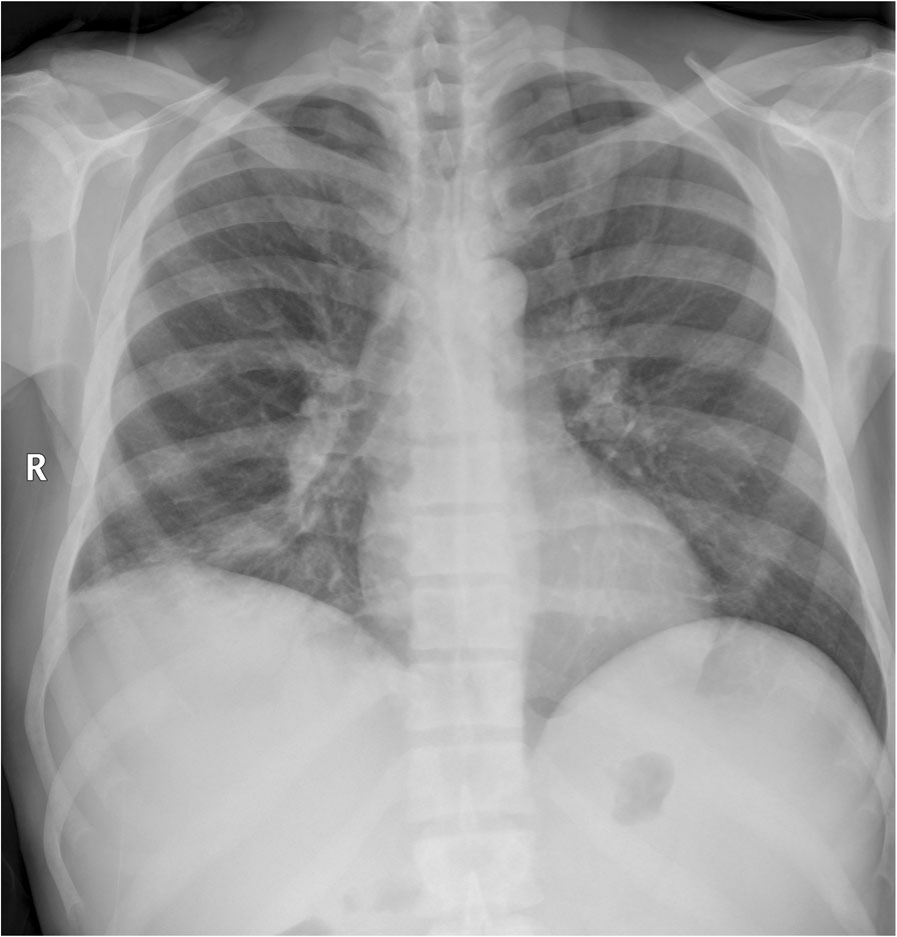
Chest radiograph demonstrating diffuse osteosclerosis in a patient with myelofibrosis. The symmetrical and diffuse appearances of osteosclerosis on plain radiographs are often subtle and difficult to identify

In PMF, osteosclerosis occurs in 30–70% of cases and is diffused, usually affecting the entire axial and appendicular skeleton [27]. A chest or abdominal radiograph may incidentally demonstrate findings of diffuse osteosclerosis, which could alert an experienced clinician to an underlying systemic disease manifesting in the bones (Fig. 13). Common differential diagnoses include endocrine causes (e.g. hyperparathyroidism), prostate cancer for an older male, and breast cancer for a female; although if coupled with massive splenomegaly, an underlying MPN is suspected.

**Fig. 14:**
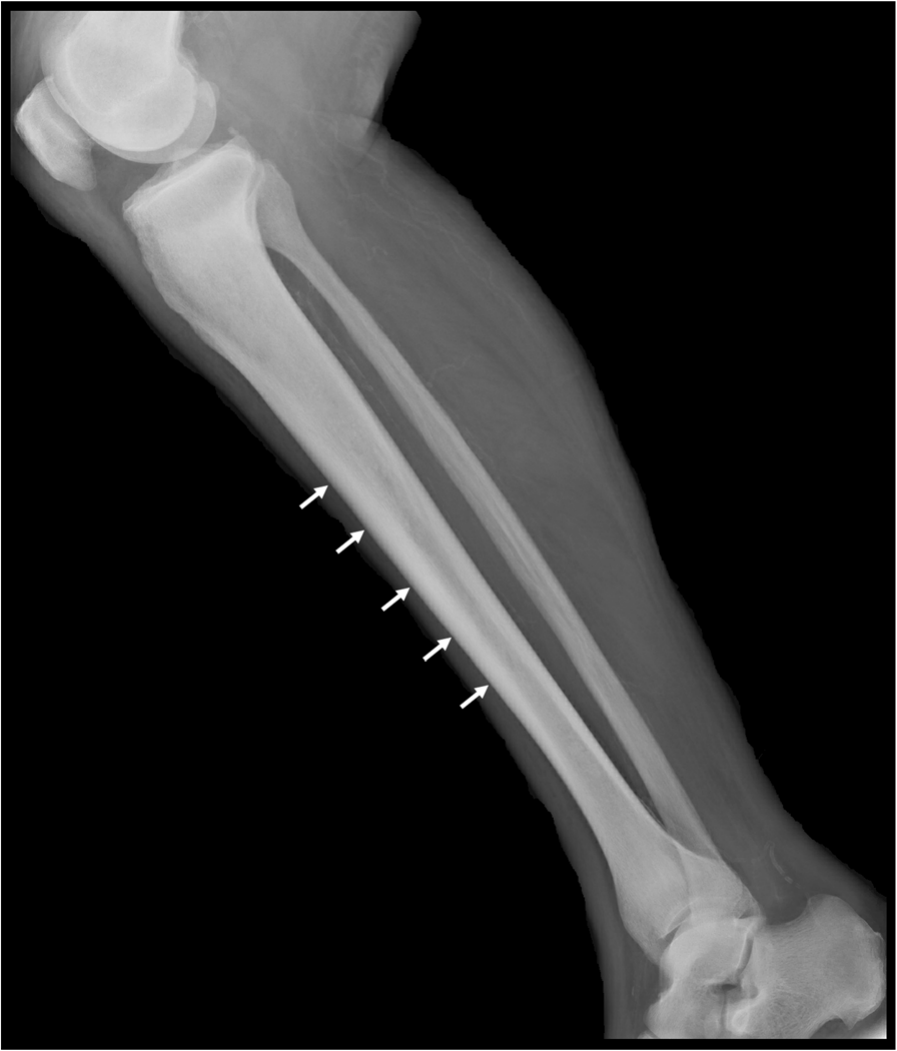
Lateral radiograph of a 62-year-old male with known primary myelofibrosis demonstrating endosteal sclerosis, the osteosclerotic pattern of myelofibrosis in the long bones (white arrows)

**Fig. 15:**
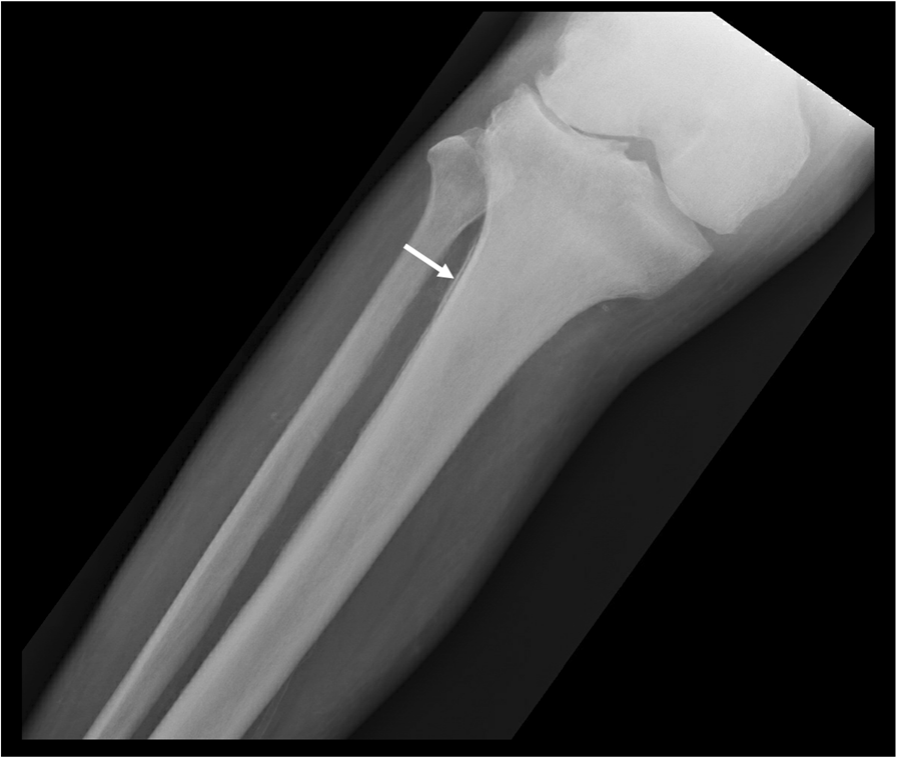
AP radiograph in the same patient demonstrates mild periostitis at the proximal tibial metaphysis, a rare feature of myelofibrosis (white arrows). Periostitis in myelofibrosis occurs at the metaphyseal regions of the distal femur or proximal tibia

**Fig. 16:**
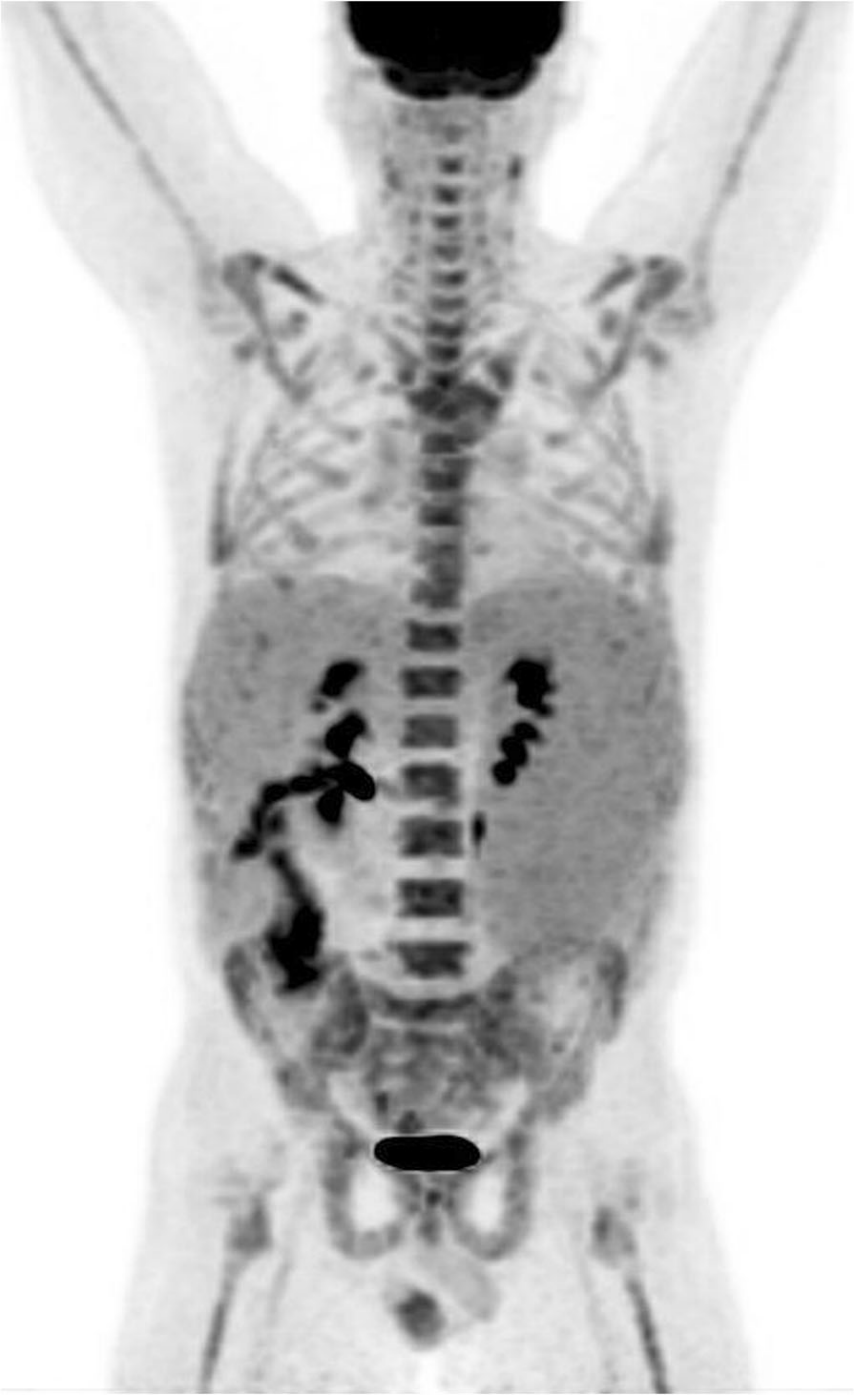
^18^F-FDG PET/CT MIP image of a patient with myelofibrosis demonstrates diffuse FDG uptake in the bones. Note the presence of hepatosplenomegaly in the patient

**Fig. 17:**
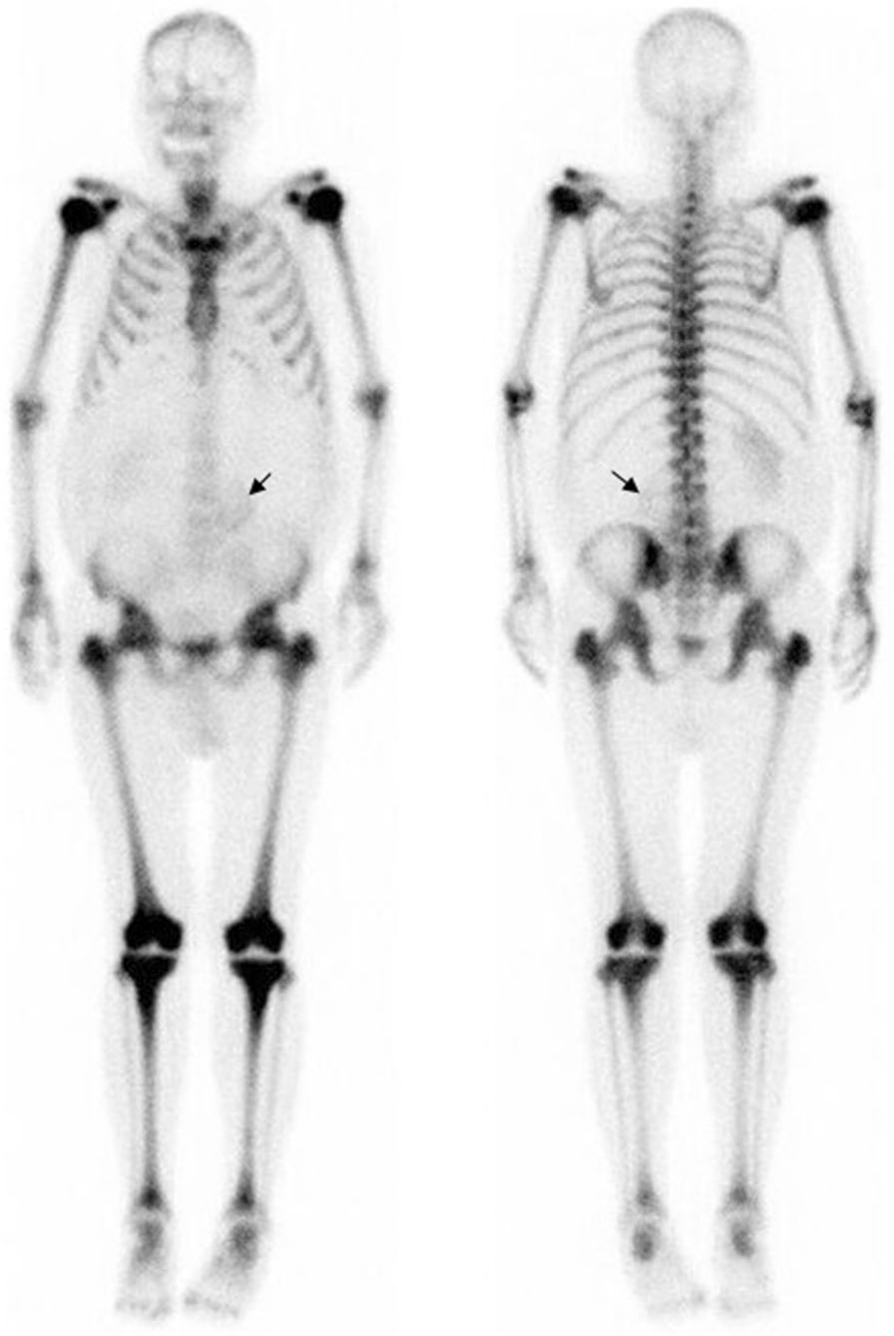
^99m^Tc Tc-99 m isotope HDP nuclear bone scan in a different patient demonstrates an appearance approaching a ‘superscan’ appearance due to intense skeletal tracer distribution. This case also provides a clue to the cause of skeletal uptake: note that the left kidney has been displaced inferiorly by an enlarged spleen from marked splenomegaly (black arrow). ^99m^Tc-99 m nuclear bone scans may be helpful in identifying marrow presence in extramedullary sites of haematopoiesis

In the long bones, cortical thickening occurs due to endosteal sclerosis (Fig. 14) [28]. Periosteal reaction is rare and when present usually occurs at the metaphyses of the distal femur and proximal tibia (Fig. 15) [29]. ^18^F-fluorodeoxyglucose positron emission tomography/computed tomography (FDG PET/CT) in PMF characteristically demonstrates intense and diffuse tracer uptake both in osteosclerotic bones and extraosseous sites of EMH, although this is a non-specific finding, and EMH in the liver and spleen is often well demonstrated on this modality especially when considerably enlarged (Fig. 16). Diffuse and intense osseous tracer uptake on nuclear bone scintigraphy with ^99m^Tc-hydroxydiphosphonate (^99m^Tc HDP) results in a ‘superscan’ appearance (Fig. 17). This is a term used on bone scan when activity in the bones is so profound that virtually, all tracer uptake becomes concentrated in the skeleton, and the usual physiological tracer concentration in the soft tissues and genitourinary tract becomes either markedly diminished or absent [30].

**Fig. 18:**
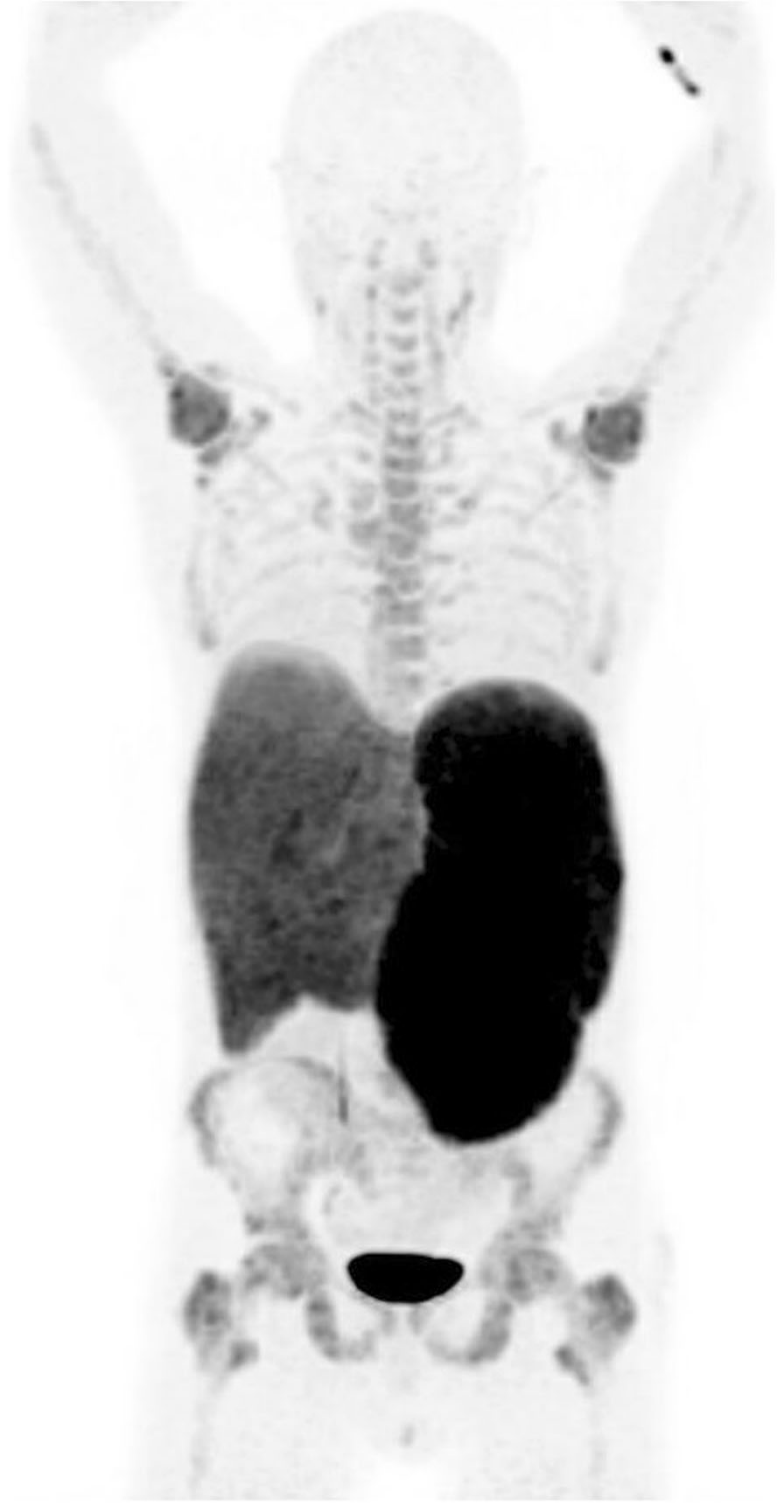
^18^F-FLT PET in a patient with myelofibrosis demonstrating diffuse increased tracer uptake in the bones and enlarged spleen. ^18^F-FLT PET is an excellent alternative to FDG due to the ability to directly assess myeloproliferative activity without the superimposed inflammatory component of disease. ^18^F-FLT PET can also be used to determine disease status or treatment outcome when performed at baseline

An excellent alternative tracer to FDG for PMF is ^18^F-fluorothymidine (^18^F-FLT PET, Fig. 18) [31]. With FDG, a more intense and widespread FDG uptake occurs at the earlier stage rather than later stage, due to the varied extent of fibrosis and inflammation present in the marrow in PMF [31]. In contrast to FDG, 18-FLT is able to directly assess myeloproliferative activity without the superimposed inflammatory component and, when performed at baseline, can be used to determine disease status or treatment outcome. In a retrospective review of patients with myelofibrosis, FLT PET/CT was found to predict response to therapy with targeted therapies (imatinib or JAK-2 inhibitors) and was also found to predict leukemic evolution [32].

**Fig. 19:**
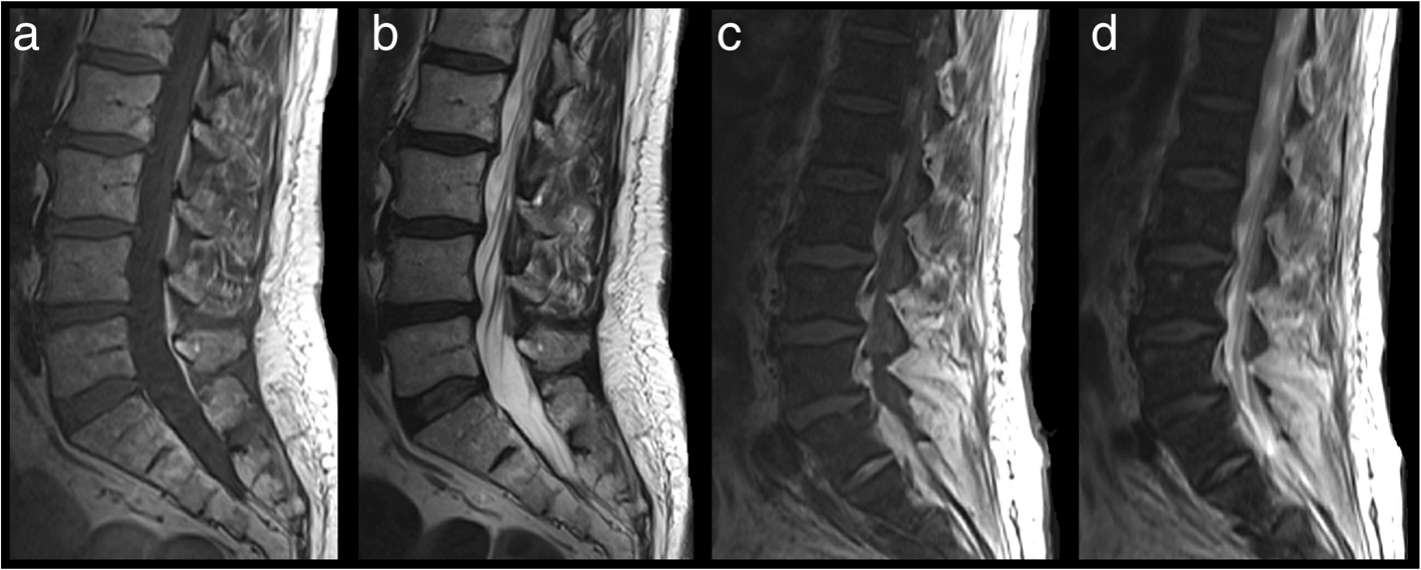
Sagittal T1 (**a**) and T2 (**b**) MRI of the lumbar spine in a normal patient demonstrating normal marrow appearances. The vertebrae are homogenous, and the intervertebral disks are hypointense relative to the marrow. In contrast, the marrow signal in myelofibrosis appears markedly hypointense on both T1 (**c**) and T2 (**d**). The intervertebral disks also appear hyperintense relative to the marrow signal

On MRI, normal marrow in healthy adults is generally more T1 hyperintense and T2 hyperintense or isointense to the intervertebral disks (Fig. 19a, b). Due to replacement of marrow fat by collagen, reticulin fibres, and cellular material [28], the T1 and T2 signal of diseased bone in PMF becomes markedly hypointense, and the marrow appears more hypointense than the intervertebral disks (Fig. 19c, d). The differential diagnosis of osteosclerosis in the spine however is wide, and differentiating PMF against the other causes of osteosclerosis can be difficult, although in some pathologies, the vertebral appearances may have classic appearances, while in others, certain extraosseous features or clue from clinical history may help establish the diagnosis (see Table 1) [33].

**Table 1:** Differential diagnosis of osteosclerosis in the spine

Differential diagnosis of sclerotic vertebral lesions			
Focal/multifocal lesions		Diffuse osteosclerosis	
Diagnosis	Helpful features	Diagnosis	Helpful features
Bone infarction	• Typically serpiginous or patchy geographic appearances.	Sclerotic metastases	• May have known history of cancer (e.g. breast, prostate, gastric, neuroendocrine).
	• The ‘Double Line Sign’ of hyperintense inner ring and hypointense outer ring is a classic feature.		• Usually solitary lesion, T1 isointense or hypointense compared to red marrow, and minimally brighter on T2/STIR.
	• H-shaped vertebrae and absence of the spleen may be a clue to sickle cell anaemia as a cause.		
			• A ‘Halo sign’ of rim hyperintensity and marked enhancement are highly suggestive.
Chronic granulomatous infection	• Often also associated with longitudinal ligament oedema and enhancement, vertebral destruction and intraosseous, and epidural and paraspinal abscesses.	Myeloproliferative neoplasms	• Diffuse, homogenous T1 hypointense but with variable T2 hypo- or hyperintensity depending on phase of disease.
			• In late myelofibrosis, depletion of haematopoietic elements results in markedly hypointense marrow appearances on all sequences.
Chronic recurrent multifocal osteomyelitis (CRMO)	• Typically children or young adults.	Sclerotic multiple myeloma	• Uncommon, occurring in 3% of myeloma cases. Appears hypointense on all sequences.
	• Clavicle involvement is a characteristic finding.		
	• Clinical features may help with diagnosis. CRMO is associated with psoriasis, inflammatory bowel disease, or skin conditions including SAPHO syndrome.		• May be associated with POEMS syndrome—clinical or radiological features of polyneuropathy, organomegaly, endocrinopathy, monoclonal gammopathy, and skin changes may be present.
Bone islands	• Generally oval-shaped and spiculated, and orientated to long axis of bone.	Osteosarcoma	• Predominantly hypointense on all sequences (T1, T2, STIR).
	• Lack bone marrow oedema, periostitis, soft tissue mass, or other aggressive features.		• Associated with large areas of new bone formation.
Lymphoma/leukaemia	• May present as a focal bone lesion or ‘ivory vertebra’ with diffuse T1 hypointense but homogenously T2 hyperintense appearances.	Lymphoma/leukaemia	• May present as a focal bone lesion or ‘ivory vertebra’ with diffuse T1 hypointense but homogenously T2 hyperintense appearances.
	• Tumour extension into soft tissues is a common feature of lymphoma.		• Tumour extension into soft tissues is a common feature of lymphoma.
	• Leukaemia more typically presents as a diffuse process rather than focal/multifocal lesion, with diffuse slight T1 hypointensity and T2 hyperintensity appearance compared to the intervertebral disks.		• Leukaemia more typically presents as a diffuse process rather than focal/multifocal lesion, with diffuse slight T1 hypointensity and T2 hyperintensity appearance compared to the intervertebral disks.
Osteoid osteoma	• Usually under 30 years of age.	Mastocytosis	• Variable appearances—may be both lytic and sclerotic, diffuse, or focal.
	• A T1 isointense and T2 hyperintense nidus is usually present in the neural arch.		
			• Typically T1 hypointense with mixed T2 and STIR signal intensity and multifocal or diffuse enhancement.
	• Clinical history is often helpful: severe pain and scoliosis, improving with non-steroidal anti-inflammatory analgesics.		• Multifocal bubbly lesions may be identified.
Osteoblastoma	• Usually under 30 years of age.	Renal osteodystrophy	• The ‘Rugger-Jersey’ appearances of T1 and T2 hypointensity along the endplates are classic findings.
	• Similar appearances to osteoid osteoma but larger (2–6 cm) and with more aggressive features (local growth and distant metastases).		
			• Renal atrophy, scarring, renal cysts, or lipomatosis may also provide clues to the underlying aetiology.
Giant cell tumour	• Usually young to middle-aged patient.	Paget’s disease	• Demonstrate fibrofatty change, trabecular disorganisation, and cortical involvement and expansion.
	• More common in sacrum than elsewhere in the spine.		
	• Usually located in the vertebral body rather than neural arch, and has heterogenous, isointense T1 signal with enhancement.		• Variable T2 appearances depending on the stage of disease.
			• The ‘Picture-Frame’ vertebra is a classic appearance at the mixed phase of disease.
	• Areas of T1 hyperintensity may be present from intralesional haemorrhage.		
	• Fluid-fluid levels may be present if associated with an underlying aneurysmal bone cyst.		
		Fibrous dysplasia	• Appears T1 isointense to hypointense and T2 hypointense. Typically a well-marginated lesion with cortical thickening, and often with a clear halo of perilesional fat on T1.
		Osteopetrosis	• Diffuse T1 and T2 hypointensity with vertebral thickening and spinal canal stenosis.
			• The ‘Sandwich Vertebra’ appearance is a classic description.
		Pyknodysostosis	• Patients often have a known history.
			• Associated with short stature and scoliosis.

## Potential complications of primary myelofibrosis

Potential complications in PMF are multifaceted and arise from progressive marrow fibrosis and ineffective haematopoiesis, complications related to EMH and acute leukemic transformation. The most common complications are thrombohaemorrhagic and vascular. Other common complications include chronic hypertension, including pulmonary and portal hypertension, and splenic infarction.

### Thromboembolic events

**Fig. 20:**
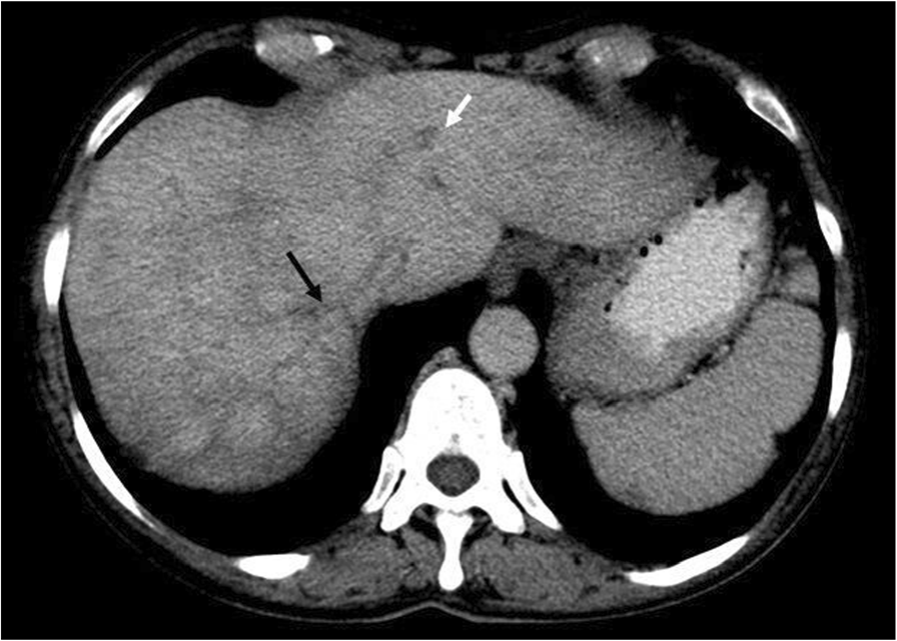
Portovenous phase axial CT demonstrating Budd-Chiari syndrome in a patient with myelofibrosis. The middle hepatic vein (black arrow) and left hepatic vein (white arrow) are occluded and non-enhancing, and the inferior vena cava (not shown) is non-opacifying and slit-like. Note the early ‘nutmeg liver’ appearance in segment 7 which is a typical feature of Budd-Chiari syndrome

**Fig. 21:**
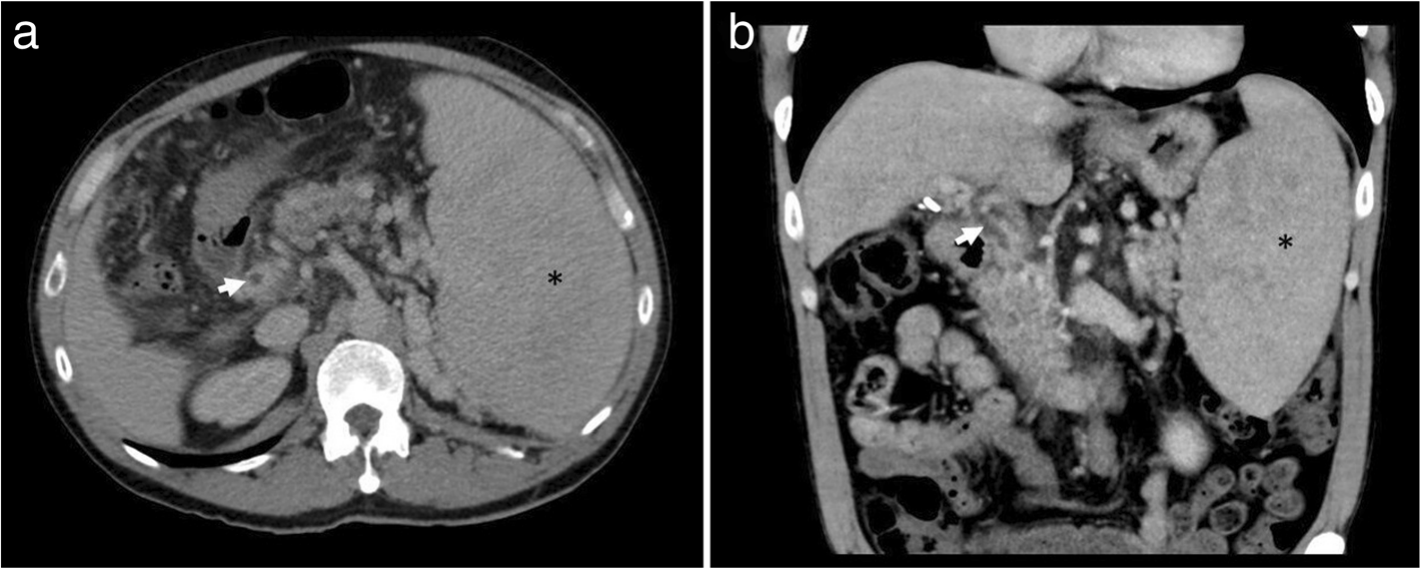
Axial (**a**) and coronal (**b**) CT demonstrating a filling defect within the portal vein (white arrows). Note the presence of splenomegaly in the patient (asterisk) and multiple splenic varices

**Fig. 22:**
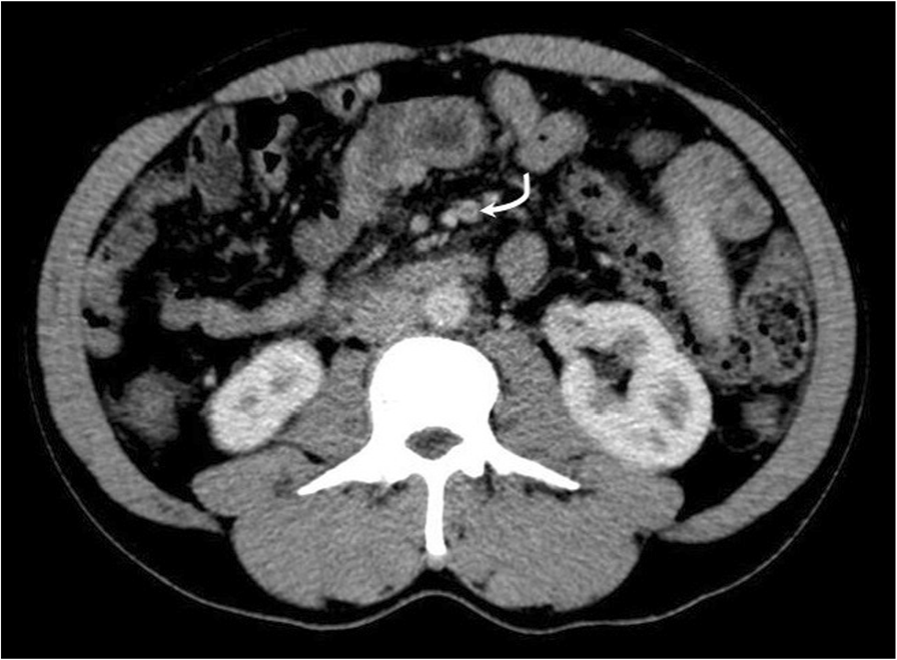
Axial CT in the same patient demonstrates a filling defect within a branch of the superior mesenteric vein. Myeloproliferative neoplasms are now recognised as the leading systemic cause of splanchnic vein thrombosis [19] and affecting a younger age group in general

**Fig. 23:**
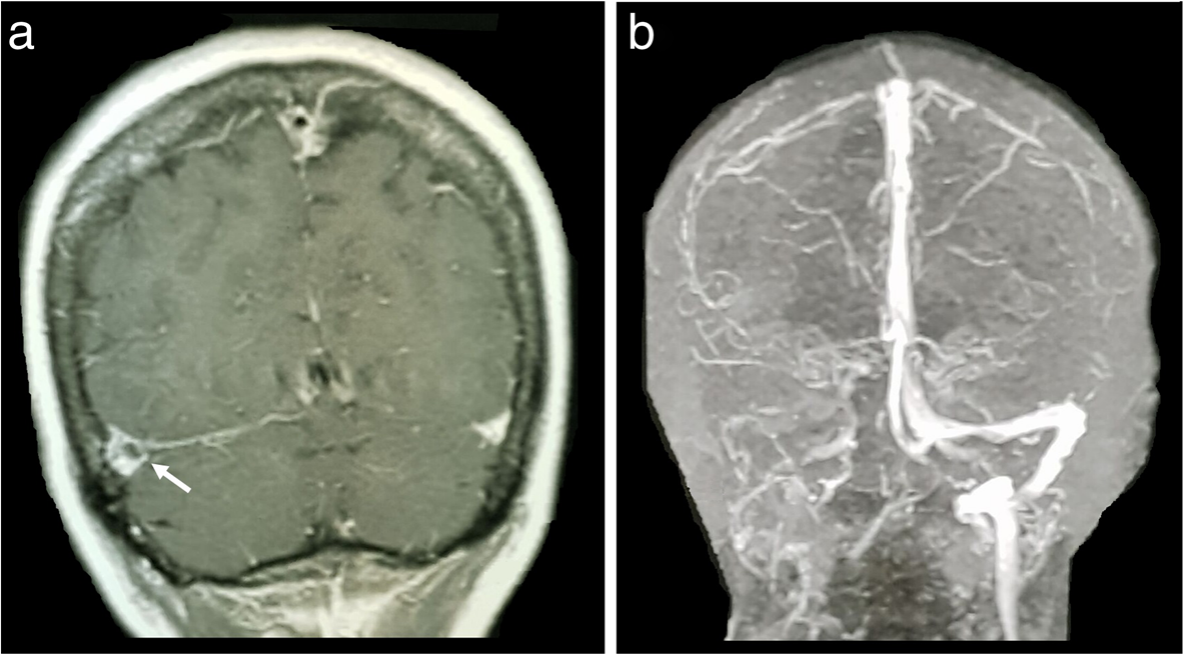
Coronal T2 MRI (**a**) demonstrating a filling defect in the right transverse sinus (white arrow). Maximum intensity projection (MIP) reconstruction of an MRI intracranial venogram (**b**) demonstrates complete non-opacification of the right transverse sinus. This was due to a chronic right transverse sinus thrombosis in a patient with myelofibrosis

**Fig. 24:**
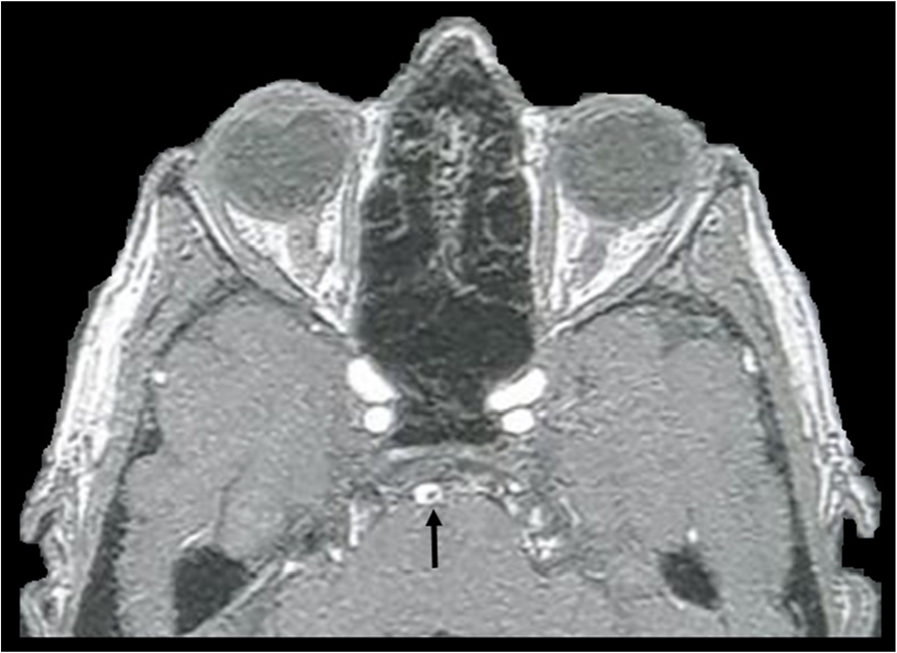
Axial CT intracranial angiogram demonstrates a filling defect within the basilar artery (black arrow) in a patient with myelofibrosis. Other multiple filling defects were seen in both vertebral arteries (not shown). Arterial embolisms are common in myelofibrosis and can result in transient ischaemic events, cerebrovascular accidents, angina or myocardial infarctions, and peripheral vascular disease

Overt thromboembolism, especially in unusual sites, is a common presentation in PMF. MPNs are now recognised as the leading systemic cause of splanchnic vein thrombosis [34] and affecting a younger age group in general. Some of the largest studies of MPN-associated splanchnic vein thrombosis studies reported a prevalence of underlying MPN in splanchnic vein thrombosis of up to 50% [35] with median age of 48 years [36]. Splanchnic vein thrombosis includes Budd-Chiari syndrome (Fig. 20), portal vein thrombosis (Fig. 21), or distal mesenteric venous thromboses (Fig. 22). Other venous thromboses include deep vein thromboses and/or pulmonary emboli and cerebral venous sinus thromboses (Fig. 23). Arterial embolisms are also common and include intracranial embolisms causing transient ischaemic events or cerebrovascular accidents (Fig. 24), angina or myocardial infarctions, and peripheral vascular disease. Thromboembolism affecting small vessels often manifests as migraine-type headaches, lightheadedness, paraesthesia, erythromelalgia, or atypical chest pains and is not often seen on imaging but is responsive to aspirin therapy [37].

The clinical management of splanchnic vein thrombosis in PMF is particularly challenging with a typically younger age of patient at diagnosis, severity of short- and long-term outcomes of inadequate treatment, and balance against the risks of haemorrhage with treatment. A recent treatment algorithm for PMF [38] advises either observation and supportive therapy or allogenic stem cell transplantation or ruxolitinib (JAK inhibitor) treatment, depending on the overall PMF prognostic score [13].

### Other complications of myelofibrosis

Progressive marrow fibrosis causes worsening cytopaenia which is itself accentuated further by pre-existing ineffective haematopoiesis. With a potentially expanded plasma volume from splenomegaly, severe anaemia can exacerbate pre-existing symptoms of tissue hypoxia in patients with vasculopathy or known cardiac failure—not uncommon in the comorbid population typically affected by PMF [19]. In thrombocytopaenia, the main risk is haemorrhage, which may be exacerbated by platelet dysfunction and concurrent varices. With leukopaenia, an increased risk of infection occurs in 22% of cases and carries a 9% mortality. The main pathogen is bacterial (78%) and usually of respiratory tract origin (52%), although viral and fungal organisms are also seen and other body systems can be a source of infection [39]. Gout can also occur due to the increased haematopoietic turnover.

**Fig. 25:**
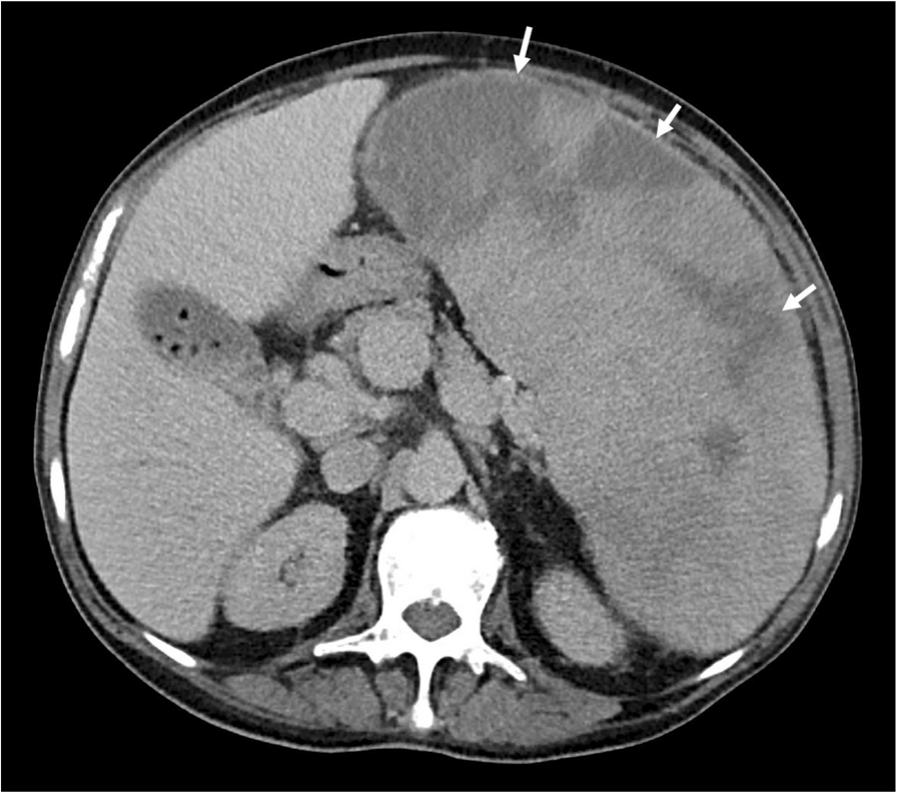
Axial portal venous phase CT of the abdomen demonstrates a markedly enlarged spleen with multiple wedge-shaped and linear areas of low attenuation consistent with splenic infarctions (white arrows). Marked enlargement of the spleen and liver may result in infarction, portal hypertension, hypersplenism, plasma volume expansion, and splanchnic vein thrombosis

**Fig. 26:**
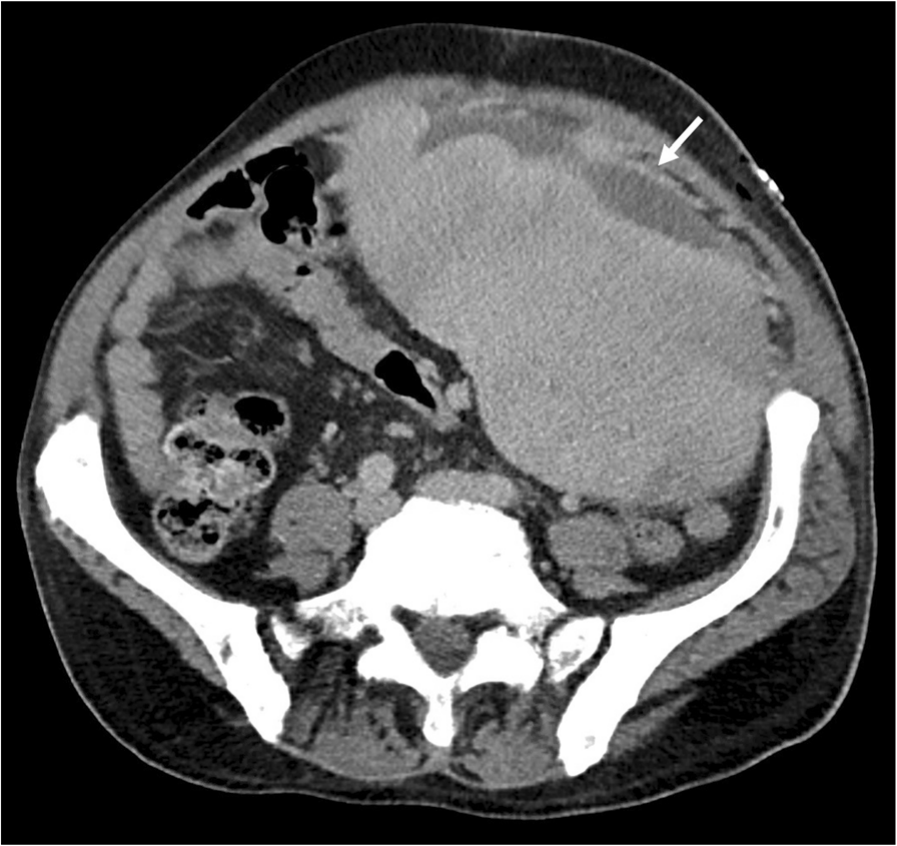
Axial CT of the abdomen demonstrates a chronic subcapsular splenic haematoma in a markedly enlarged spleen (white arrow). Severe organomegaly increases the risk of organ rupture with minor trauma or even spontaneously

As described above, EMH occurs in a variety of organs, most commonly in the liver and spleen. Marked enlargement of the spleen and liver may result in infarction (Fig. 25), mass effect symptoms, portal hypertension (Fig. 6), hypersplenism, plasma volume expansion, and splanchnic vein thrombosis (Figs. 20, 21, and 22). Severe organomegaly increases the risk of organ rupture with minor trauma or even spontaneously (Fig. 26). Atraumatic splenic rupture in general is rare but occurs most commonly in malignant haematological neoplasms [40]. Portal hypertension affects 7% of patients, due to increased hepatic blood flow, intrahepatic venous obstruction, and stasis with splenomegaly [41]. Hepatomegaly occurs in 39–65% of patients [19].

## Summary

PMF is a disease with classic imaging appearances but also has complex appearances in advanced disease where there are several potential multi-organ complications. An understanding of the underlying disease process in PMF-associated osteosclerosis and EMH knowledge of the related complications especially splanchnic vein thromboses and other unusual thromboembolic locations allows for easier identification and differentiation of these disease-related complications from a wider differential diagnosis.

## Data Availability

Data sharing is not applicable to this article as no datasets were generated or analysed during the current study.

## Abbreviations

CML: Chronic myeloid leukaemia
EMH: Extramedullary haematopoiesis
FDG PET/CT: ^18^F-fluorodeoxyglucose positron emission tomography/computed tomography
MPN: Myeloproliferative neoplasm
PMF: Primary myelofibrosis
